# Formation of Carcinogens in Processed Meat and Its Measurement with the Usage of Artificial Digestion—A Review

**DOI:** 10.3390/molecules27144665

**Published:** 2022-07-21

**Authors:** Ewelina Pogorzelska-Nowicka, Marcin Kurek, Monika Hanula, Agnieszka Wierzbicka, Andrzej Półtorak

**Affiliations:** Institute of Human Nutrition Sciences, Department of Technique and Food Product Development, Warsaw University of Life Sciences (WULS-SGGW), Nowoursynowska 159 C Street, 02-776 Warsaw, Poland; marcin_kurek@sggw.edu.pl (M.K.); monika_hanula@sggw.edu.pl (M.H.); agnieszka_wierzbicka@sggw.edu.pl (A.W.); andrzej_poltorak@sggw.edu.pl (A.P.)

**Keywords:** artificial digestion, in vitro digestion, meat, carcinogens, heterocyclic aromatic amines (HAAs)

## Abstract

Meat is a rich source of various nutrients. However, it needs processing before consumption, what in turn generates formation of carcinogenic compounds, i.a., polycyclic aromatic hydrocarbons (PAH), nitrosamines (NOCs), and the most mutagenic heterocyclic aromatic amines (HAAs). It was widely found that many factors affect the content of carcinogens in processed meat. However, it has recently been discovered that after digestion free HAAs are released, which are not detectable before enzymatic treatment. It was established that the highest percentage of carcinogens is released in the small intestine and that its amount can be increased up to 6.6-fold. The change in free HAAs content in analyzed samples was dependent on many factors such as meat type, doneness, particle size of meat, and the enzyme concentration used for digestion. In turn, introduction of bacteria naturally occurring in the human digestive tract into the model significantly decreases total amount of HAAs. Contrary, the addition of food ingredients rich in polyphenols, fiber, and water (pepper powder, onions, apples) increases free HAAs’ release up to 56.06%. Results suggests that in vitro digestion should be an integral step of sample preparation. Artificial digestion introduced before chromatographic analysis will allow to estimate accurately the content of carcinogens in processed meat.

## 1. Introduction

Meat (especially red meat) should play a significant role in the human diet, as it contains many nutrients. The Food and Drug Administration [1] recognized meat as an excellent source of protein (“High in/Rich in/Excellent”). The US Department of Agriculture set the percent daily value (DV) for beef and pork proteins at 40%, for lamb at 37%, while for veal and game at 36% [2]. Furthermore, meat proteins are believed to elevate utilization of vitamin D, particularly when low exposure to sun occurs. Meat consumption also provides 30–35% of the daily requirement for vitamin D and 21% for iron. Those are two out of five shortfall nutrients (vitamin D, calcium, potassium, iron, and fiber) in human diets that were indicated in the US Dietary Guidelines for Americans as posing a serious health concern for society [3,4]. Beef, pork, veal, and lamb also contain potassium, a third shortfall nutrient in the human diet, in amounts that qualify to label them with a “source of potassium” claim (at least 15% of the EU Nutrient Reference Value in 100 g of meat). Meat (beef, pork, lamb, veal) is also a “source of” or “high in” other nutrients such as: phosphorus, selenium, niacin, pantothenic acid, vitamin B6, and vitamin B12 [2]. Moreover, Cosgrove et al. [5] reported that people who consume meat at higher levels have a lower risk of zinc, vitamin C, and riboflavin deficiencies. 

However, at the same time, according to the International Agency for Research on Cancer (IARC), consumption of red meat is probably carcinogenic to humans [6]. It has been established that the consumption of processed meat 50 g over the recommended level increases the probability of cancer occurrence by about 18%. There are several factors responsible for the detrimental effect of meat consumption on human health. Most of them are linked to its processing: cooking, excessive salt addition, curing, and smoking. Cooking of meat improves its digestibility and sensory value but most importantly eliminates microorganisms which pose serious health risks for humans [7]. It has been proved that cooking of meat to a temperature of 70 °C in the core and keeping it for two minutes ensures its microbiological safety [8]. Nonetheless, high temperatures during the treatment lead to the formation of heterocyclic aromatic amines (HAAs) and polycyclic aromatic hydrocarbons (PAHs), potential and proven carcinogens. Curing and smoking are responsible for the formation of carcinogenic substances such as N-nitroso-compounds (NOCs) and polycyclic aromatic hydrocarbons (PAHs).

The final content of PAHs and HAAs in meat is determined by many process- and material-related factors such as: temperature, time, type of meat (beef, veal, pork, poultry), quality of meat, shape and size of meat, grinding degree, type of heat treatment (frying, grilling, roasting, etc.), pH, precursor content (mainly amines and hexoses content), water content, content of divalent ions, fat content, and fat oxidation state. Moreover, the addition of bioactive compounds to meat may also affect the formation of carcinogens. Polyphenols and flavonoids are bioactive compounds, most commonly used for meat enrichment. Those chemicals are widespread in nature and might be found, for instance, in herbs, spices, seeds, and other plant materials. Numerous epidemiological studies have reported their protective effect against developing human diseases such as: ischemic heart disease, diabetes, or even cancer. Those properties were linked with their antioxidant activity. Polyphenols may also exert a positive effect on human health, indirectly. For instance, bioactive compounds inhibit formation of carcinogens in meat during heat treatment. Depending on the existing conditions, the content of carcinogenic substances can change even 100 times [9].

In most studies performed to date, the content of unbound molecules was measured. In 2013, Szterk [10] reported that the substantial share of carcinogens is released from the meat matrix only after digestion. Those compounds were not detected in samples before enzymatic treatment. Thus, data gathered to this day do not give complete knowledge on the effect of process parameters and the addition of bioactive compounds tested before on the total amount of carcinogens in heat-processed meat. Therefore, we decided to review the impact of factors affecting the content of HAAs and PAHs. In the second step, we gathered information describing the change in carcinogens content after artificial digestion. This exhibits how inevitable it is to perform digestion in order to estimate HAAs and PAHs content properly. Summarizing, the review underlines how many factors need to be re-tested using an artificial digestion as a sample preparation step to obtain accurate information about their effect on total content of carcinogens (free and bound) in heat-treated meat.

## 2. Thermally Processed Meat Is a Source of Carcinogenic Compounds

Heterocyclic aromatic amines are a group of compounds with a structure consisting of 2–5 aromatic cycles, 1 or more nitrogen atoms, and 1 exocyclic amino group (with the exception of harman, norharman, and Lys-P1) [11]. HAAs are formed in products with high protein content during heat treatment exceeding a temperature of 120 °C. The main substrates for HAAs formation are free amino acids found in muscle tissue, especially creatine and creatinine. Taking into account that HAAs are produced during Maillard reactions, the degree of their formation also depends on the pH of the meat. Over 25 HAAs were isolated and identified in thermally processed food in the past 40 plus years. HAAs might be divided into two large groups in terms of their polarity. Polar HAAs (amino-imidazo-azarens, IQ group) are formed during food processing in high temperatures of 100–300 °C (smoking, frying, toasting, grilling). Pyroindols and pyro imidazoles are non-polar HAAs which form also during heat treatment of food but at temperatures above 300 °C; therefore, they are called pyrolytic amines. HAAs are probably the most mutagenic and carcinogenic compounds generated in heat-treated food products. It has been reported that they are 2000-fold more mutagenic in comparison to benzo[a]pyrene and 100-fold stronger than aflatoxin [12,13]. Nine heterocyclic aromatic amines (including MeIQ, MeIQx, PhIP, AαC, MeAαC, Glu-P-1, Glu-P-2, Trp-P-1, Trp-P-2) have been recognized by the IARC as possible human carcinogens (group 2B) and one (2-amino-imidazo [4,5-ƒ]quinoline) as a probable carcinogen (2A group). It has been proved that HAAs can induce tumors in rodents and primates (nonhuman) at several locations such as: the lungs, liver, mammary glands, prostate, and colorectum [14]. In the United States, the National Toxicology Program listed four HAAs (PhIP, IQ, MeIQ, 8-MeIQ) as carcinogenic to humans.

Polycyclic aromatic hydrocarbons are pollutants containing two or more fused aromatic rings. They are formed as a result of incomplete combustion of organic matter. PAHs include many harmful components of carcinogenic, mutagenic, and teratogenic nature with benzo(a)pyrene being the most predominant [15]. They are commonly spread in nature (so far 100 compounds are recognized). However, humans are exposed to them mainly through food (>90% of exposure). Cooking methods such as grilling, roasting, smoking, and barbecuing generates PAHs formation. Even though PAHs can be formed also from proteins and carbohydrates, the rapid increase in its content is observed during the fat pyrolysis at temperatures above 200 °C. Many factors affect PAHs formation in cooked meat, such as: temperature, cooking time, fat content, type of fuel used for cooking, and the occurrence of melted fat dropping onto the heat medium. The International Agency for Research on Cancer of the World Health Organization classified three PAHs (benz[a]anthracene, benzo[a]pyrene, dibenz[a,h]anthracene) as probably carcinogenic for humans (2A group) and three (benzo[a]fluoranthene, benzo[k]fluoranthene, indeno[1,2,3-cd]pyrene as possibly carcinogenic to humans (2B group). It is worth noting that according to the EFSA the acceptable level for benzo[a]pyrene is 2 μg/kg product, while the sum of the four PAHs bezno(a) pyrene (BaP), chrysene (CHR), benzo(a)anthracene (BaA), and bezno(b)fluoranthene (BbF) must not exceed 12 μg/kg [16].

In terms of NOCs, N-nitrosodimethylamine (NDMA) is the most carcinogenic, as a dose of 10 µg/kg body weight is sufficient for mouse feed. This is a component that directly depends on the nitrogen concentration in the meat; hence, the amount is significantly higher in cured foods. For example, in the shoulder it is of about 2.46 µg/kg NDMA, while for the cured shoulder it is over 9 µg/kg. The highest NDMA values are observed in smoked products ranging between 10 and 17 µg/kg, while the lowest values of around 1–2 µg are observed for pasteurized canned products [17]. NDMA is also observed in products other than beef and pork. Very high amounts are found in salted fish—12.64–322.92 µg/kg, which is a very big difference compared to fresh fish, where the content of this substance ranges from 0.04 to 3.5 µg/kg. Quite low NDMA values are observed in poultry, as chicken contains about 0.52, while duck meat contains 0.13 µg/kg [18]. The content of NOCs is directly dependent on the heat treatment method used (smoking, steaming) and the curing process.

It is also worth mentioning that ingredients typically found in meat products are not the only ones responsible for the formation of carcinogenic compounds. Substances that are air pollutants, such as toxic trace elements, polychlorinated dibenzo-p-dioxins and dibenzofurans, polychlorinated biphenyls, polybrominated diphenyl ethers, polychlorinated diphenyl ethers, polychlorinated naphthalenes, and perfluoroalkyl substances can also constitute a substrate for the formation of carcinogenic compounds during heat treatment [19].

Because of the potential for the formation of carcinogenic compounds in processed meat, many nutrition organizations recommend limitation of its consumption. For example, the UK NHS in its recommendations created by the Department of Health and Social Care takes the position that a maximum intake of 90 g of meat per person per day is safe, but the efforts should be made to reduce this intake to 70 g per day. This is because the previous limit was based on a 1998 consensus by the Committee on Medical Aspects of Food and Nutrition Policy [20]. Moreover, the International Agency for Research on Cancer recommended a fairly similar level, saying to reduce the consumption of processed meat to less than 55 g per person per day what is an equivalent of, for example, four slices of bacon [21]. In turn, health agencies in Australia recommend to limit the red meat intake to 455 g and to exclude from the diet processed meat products such as Frankfurters, salami, bacon, and ham [22].

## 3. Parameters Affecting HAAs Content in Cooked Meat

### 3.1. Cooking Parameters Affect Content of Carcinogenic Compounds in Meat

Thermal conditions are crucial factors which directly affect HAAs and PAHs content in meat [23,24]. The speed of HAAs and PAHs formation accelerates with the temperature increase [25]. Steaming, boiling in water, or heating in a microwave, are cooking methods that operate at low temperatures usually not exceeding 100 °C. HAAs are not detected or detected at scarce amounts in meat prepared under such conditions [26,27]. It has been established that mutagenic substances start to form at 125 °C [28]. Further, the dynamics of HAA formation increase significantly at 150 °C, while the highest increase in the content of these mutagenic compounds is observed at 200 °C. In pork fried at 125 °C, 58 ng/g of HAAs was detected while at 150 °C almost two-fold more (91 ng/g of HAAs). Similarly for beef meat, the amount of IQx, MeIQx, PhIP, and 4,8-DiMeIQx generated during thermal processing increased 2.5, 6, and 8 times, respectively, when the temperature rose from 170 to 200 °C [29]. In turn, PAHs start to form at 200 °C and the highest amount is observed after the temperature rises to 500–700 °C. The type of cooking method is the next important factor determining mutagenic activity of heat-treated meat [30,31]. Different cooking methods have different effects on the content of carcinogenic compounds. For instance, grilled drake meat is characterized by the lowest HAAs concentration in comparison to pan-fried, roasted, and deep-fried meat [32]. The source of energy also matters. Chicken grilled with charcoal had a higher content of PAHs than oven-grilled chicken [33]. Generally, it was established that more gentle cooking methods (operating at lower temperatures such as boiling and roasting) generate less mutagenic compounds [11]. Analogously, cooking methods operating at high temperatures (pan frying, grilling, oven roasting) lead to formation of mutagens at higher levels [11]. This trend was observed in various studies. However, temperature itself is not only factor that determines HAAs content in meat. An example of that was found in a study where stone-barbecued beef steak was characterized by a higher amount of HAAs in comparison to wire-barbecued beef steak. The results suggested that there are other important factors such as the type of heat transfer (conduction, convection, radiation) and occurrence of surrounding media (water, fat, metal, air) which changes heat transfer coefficients [34]. The coefficient is better in methods with direct contact. For pan frying, the coefficient is equal to 150 Wm^2^/K in comparison to 30–40 Wm^2^/K for air convection. That is why the meat prepared on a contact grill had 10 times higher MeIQx content than meat prepared using a convection oven and deep-fat fryer [35]. In the case of oven roasting, heat is transferred indirectly by convection and similarly for microwave heating where heat is transferred indirectly by radiation. Thus, the meat cooked in a microwave contains much fewer carcinogenic compounds in comparison to deep-fried or charcoal-grilled beef and chicken [36]. In the case of direct heating, for instance by pan frying, there is a possibility to reduce HAAs formation by turning the meat in the pan, frequently. It is also extremely important not to let the melted fat fall onto the heat source. The direct contact of fat with the flame results in the formation of a huge amount of PAHs. PAHs content is influenced by both source of the product and the method of thermal processing. However, the latter is of greater importance. For example, benzo[a]pyrene content was revealed to be 0.7 µg/kg for smoked pork meat and 1.3 µg/kg for chicken meat. In the case of sausages subjected to three processing methods, smoked sausage contained 1.1 µg/kg of benzo[a]pyrene, grilled sausage 0.5 µg/kg, and cooked sausage 0.15 µg/kg [37]. However, those factors mentioned above (temperature and cooking method) occur simultaneously and thus it is important to verify their additive impact on the final content of HAAs, what we present in Table 1.

**Figure 1:**
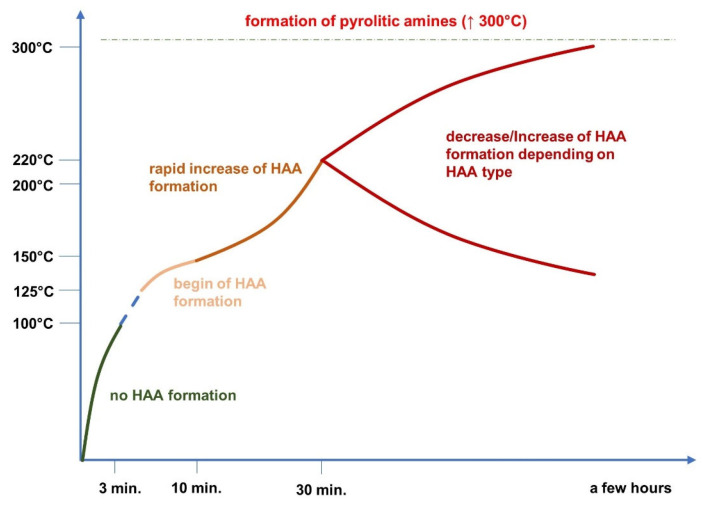
Changes in the content of HAAs depending on applied temperature and time.

Time also exerts a significant impact on the formation of carcinogenic compounds. Measuring the aqueous model system of meat extracts, it was noted that the content of 4,8-DiMeIQx after heating (175 °C) for one hour was 1.2 ng/g, when the time was doubled (at the same temperature) the amount of 4,8-DiMeIQx increased three times [48]. The same trend was observed in a study analyzing the HAAs content in ground beef fried on the pen at 150–180 °C. The small level of those substances was found after 3 min of the process; however, the HAAs amount increased with time passage [49]. In the case of beef there are studies documenting that the HAAs do not form in less than five minutes of heat treatment, and certain mutagens (IQ, MeIQ, 4,8-DiMeIQx, AαC, and harman) were detected in a small amount in no less than 10 minutes. However, the impact of time on the HAAs formation is directly linked with temperature of the process (Figure 1). In, turn, PAHs formation is most pronounced during the first few minutes of cooking. It is probably caused by its deposition on the meat surface what cuts off the fat (precursor) from the heat medium and in turn further formation of PAHs is not possible [50].

Generation of HAAs would be impossible without precursors: creatine, carbohydrates, amino acids, nitrogenous bases, and nucleosides. Creatine under heat treatment converts into the physiologically inert product creatinine. In turn, creatinine is essential during formation of the imidazole ring. Thus, formation of some HAAs such as IQ and IQx is completely dependent on creatinine content in meat [51]. This process is graphically depicted in an article by Cao et al. [52]. It was proven that beef containing a low level of creatinine was less mutagenic than beef with a high creatinine content [53]. However, formation of non-polar HAAs is not affected by creatinine content. Thus, other amino acids are necessary to create HAAs as well. Different amino acids can be precursors for the formation of the same mutagenic compounds, e.g., glycine, serine, threonine, lysine, and alanine, contribute to the formation of MeIQx [54]. Equally important precursors for HAAs formation are reducing sugars (glucose, fructose, lactose, ribose). However, the key is the ratio between the content of amino acids and sugars. When the content of sugar is higher than in the natural state (half the molar quantity of sugar to the amount of amino acids) the HAAs generation is reduced [55]. Thus, there was a 50% decrease in HAAs content observed in meat obtained from pigs with the RN allele (higher glycogen concentration) in comparison to meat acquired from normal pigs [56]. In general, the content of HAAs varies significantly depending on the meat type what is caused by the differences in the content of precursors. For instance, in ground beef, 50 ng/g of PhIP was detected [57] and 2 ng/g of MeIQx. Slightly lower values for PhIP were obtained when meat was fried at 200–230 °C reaching the level of 10–25 ng/g. Interestingly, significantly higher values are observed for poultry meat. For instance, in barbecued breast fillets the PhIP content reached 480 ng/g when in oven-broiled meat only 72 ng/g of PhIP was measured [58]. The lowest HAAs values were observed for pork. Grilled pork steaks contained about 28.62 ng/g of PhIP [59].

The fat content and its oxidative status was proven to have an impact on the formation of mutagenic substances as well. The more efficient ability of fat to transfer heat enables to reach higher temperatures in a relatively short time, what increases generation of HAAs and PAHs in meat. Furthermore, higher fat content reduces the proportion of other precursors needed for HAAs formation such as creatine, sugars, and amino acids. It has been proved that increased level of fat above the optimal ratio decreases HAAs content [60]. Knize et al. [61] have reported that ground beef containing 30% of fat was less mutagenic (150,000 revertants per kg of fresh beef) than beef containing 15% of fat (230,000 revertants/kg). Moreover, the oxidative status of fat is equally important. The oxidized fat increases the level of carcinogenic compounds in tested samples [62].

As far as the sum of PAHs in meat products is concerned, its content depends mainly on the smoking method as well as on the wood used for the process. For example, the sum of PAHs (BaP, BaA, BbF, CHR, and BjF (bezno(j)fluoranthene) in pork smoked on plum wood chips was 221 µg/kg, while that smoked on apple wood chips was 27.3 µg/kg [63]. The selected factors affecting heterocyclic aromatic amines content and/or mutagenic activity of samples are described in Table 2.

### 3.2. Bioactive Compounds Are Effective Inhibitors of Carcinogen Formation in Heat-Treated Meat

An effective method to reduce the formation of HAAs is to enrich meat with substances of antioxidant properties because radicals may take part in the mechanism of HAAs formation and Maillard reaction. It was proved in an electron paramagnetic experiment where the authors observed that antioxidants inhibit radical reactions which take part in HAAs generation [73]. To date, over 100 types of different substances have been tested for possible usage in the inhibition of HAAs formation. One of them was vitamin E. It was shown that vitamin E can efficiently reduce the IQ formation in meat [64]. The addition of vitamin E in a concentration of 1–10% directly onto the meat surface decreased HAAs content in fried beef patties in the range from 45% to 75%. Similar results were observed for meat obtained from cows fed with an increased level of vitamin E. In this meat, a smaller number of mutagenic compounds after cooking were measured [65]. Nonetheless, there are also studies documenting that vitamin E may increase the formation of HAAs in meat [74]. 

Carotenoids slow down the production of carcinogenic substances in heat-treated meat as well. Addition of tomato extract (1000 mg/kg) to meat juice inhibited MeIQx formation by about 13% and 4,8-DiMeIQx formation by 5% [66]. What is interesting, the same extract suppressed generation of those compounds in a chemical model system to a greater extent (36% for MeIQx and 11% for 4,8-DiMeIQx). Thus, the chemical model system is not an optimal way to assess the inhibitory effect of bioactive compounds on the HAAs formation in real food. Similarly, extracts of other fruits and vegetables rich in carotenoids and xanthophylls (carrots, oranges, apricots, Brussels sprouts, peppers, and tomatoes) were found to be effective in terms of decreasing bacterial mutagenic activity caused by IQ by about 27% (in vitro studies) [75]. 

Water-soluble vitamins may also decrease the production of carcinogens in food. A study performed by Wong et al. [67] revealed that 6 (ascorbic acid, nicotinic acid, biotin, thiamine, pyridoxamine, and pyridoxine) out of 11 tested water-soluble vitamins (ascorbic acid, vitamin B1(thiamin), vitamin B2 (riboflavin), vitamin B3 (nicotinic acid), vitamin B5 (panthotenic acid), vitamin B6 (pyridoxamine), vitamin B6 (pyridoxine), vitamin B6 (pyridoxal), vitamin B7 (biotin), vitamin B9 (folic acid), and vitamin B12) exhibited an effect against HAAs formation. Pyridoxamine (40%) was the most potent followed by niacin and ascorbic acid (20%). The effectiveness of vitamins was not correlated with their antioxidant potency. It was rather related with their capacity to trap main intermediate compounds for HAAs formation. For instance, pyridoxamine was found to neutralize phenylacetaldehyde, the main compound identified for PhIP formation. 

Furthermore, other substances such as tartaric acid obtained from tamarind suppresses the creation of carcinogens in thermally processed meat as well. It was observed that the addition of turmeric decreased harman and norharman content by almost twofold [76].

Nonetheless, polyphenols are the largest group of antioxidants that elongate the shelf-life of meat [77], elevate its healthiness [78], and decreases carcinogen formation in thermally processed meat. There are many studies documenting the effect of various natural sources of polyphenols such as: *Rosa rugosa* tea extract [79], grape seed and rosemary extract [80], green tea extract [81], artichoke extract [82], apple peel extract [68], pomegranate seed extract [83], and hawthorn extract [84] on HAAs content in meat. Depending on the extract type, inhibition of individual or total HAAs formation was up to 100%. The method of extract distribution greatly affects its inhibition capability against HAAs generation. It has been reported that spreading 0.3% of apple peel extract on the surface (marinating) was much more effective (83% inhibition of PhIP) than mixing the same amount of extract in whole volumes of beef patties (60% inhibition of PhIP) [68]. Time of meat marination also affects the final content of carcinogens in heat-treated meat. Quelhas et al. [85] have observed that the content of HAAs in meat decreased along with the length of the marinating time. Herbs and spices due to the content of various bioactive compounds i.a., polyphenols also exhibit an inhibitory effect against HAAs formation. For instance, turmeric at 5% concentration decreased harman content by about 94.8% while norharman by about 49.56% [69]. Similar effects were documented for red pepper [70], Sichuan pepper, black cumin [86], rosemary, turmeric, galangal, fingerroot [87], thyme, savory, and oregano [88]. However, not every spice and herb decreases the HAAs content in meat products. Some of them, such as fennel, anise, chili, and black pepper even promote HAAs (DMIP, PhIP, MeIQx, 4,8-DiMeIQx) generation in meat (beef patties) [89]. Nonetheless, some other studies showed inhibitory effects of black pepper or chili pepper towards formation of carcinogens in meat [90,91]. Those contradictions may arise from the fact that the inhibitory effects of herbs and spices depends on the presence and share of individual active substances contained in them, the effects of each can mutually neutralize or intensify. For instance, there are studies documenting the inhibitory effect of pure phenolic compounds (ferulic acid, quercetin, protocatechuic acid, chlorogenic acid, p-coumaric acid, quercetin, luteolin, naringenin, and rutin) in terms of suppressing the generation of HAAs in meat [71,92,93]. Some of those compounds mitigate formation of carcinogenic compounds up to 100%. However, there are also phenolic compounds such as carnosic acid and rosmarinic acid which elevate HAAs formation in chemical model systems [94]. Zeng et al. [92] who compared six pure phenolic compounds (ferulic acid, chlorogenic acid, protocatechuic acid, *p*-coumaric acid, rutin, and luteolin) observed increased content of HAAs in roasted beef patties enriched with pure phenolic acids. The authors mixed them with each other to mimic the composition of selected herbs, and stated that the mixtures of those phenolic compounds needed to be tested rather than single compounds in order to accurately estimate their effect on HAAs level in meat. Moreover, it has been proved that the antioxidant potential of polyphenols is not correlated with their impact on the final content of carcinogens in meat [71]. Studies on 25 phenolic compounds revealed that the most effective inhibitors of PhIP formation were polyphenols that contain two hydroxyl groups at *meta* positions in aromatic rings [95]. Substitution of those with carboxylic or alkyl groups reduces their inhibitory effect [96]. In turn, addition of hydroxyl and amino groups completely reduces the inhibitory effect of polyphenols. Similarly, *ortho* and *para* dihydroxy derivatives were not as effective as PhIP inhibitors. Moreover, alcohol promotes the inhibitory effect of polyphenols against HAAs generation. Viegas et al. [72] have reported that marination of beef with mixtures containing wine and herbs was more effective against (73.5%) heterocyclic aromatic amines formation in comparison to marination with dealcoholized wine and herbs (53.4%).

## 4. Digestion Significantly Increases the Number of Total Carcinogens Detected in Heat-Treated Meat

**Figure 2:**
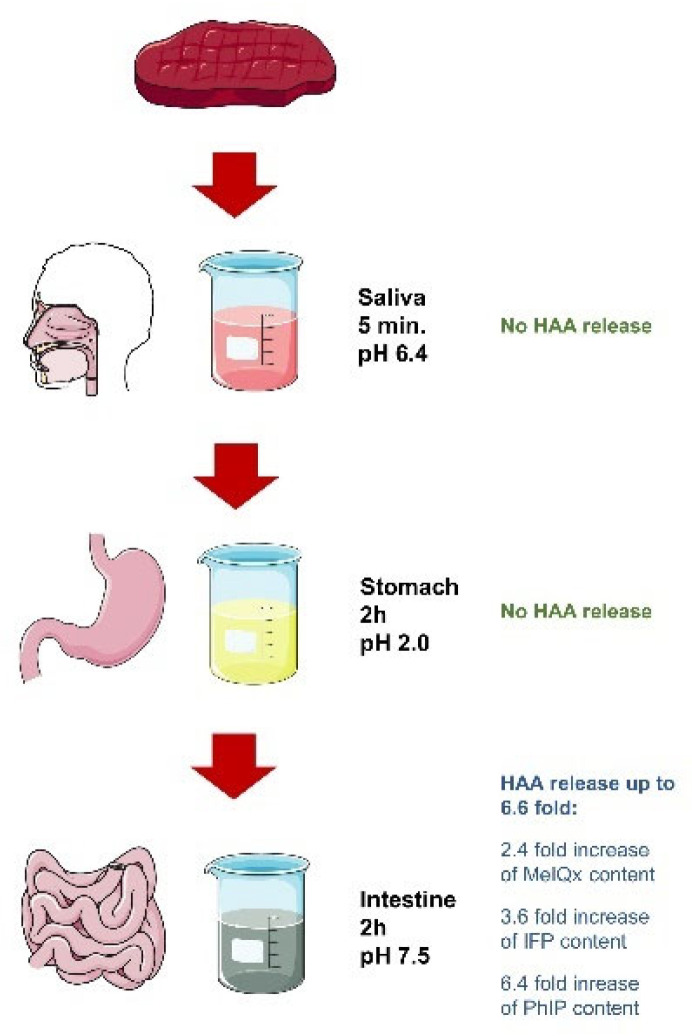
Release of HAAs at each stage of the artificial digestion tract.

The studies on the content of heterocyclic aromatic amines and polycyclic aromatic hydrocarbons concern almost exclusively the content of free compounds. However, due to high temperature, HAAs can be adsorbed on the surfaces of polymeric structures such as proteins or glycogen. Moreover, HAAs can bind with peptides and proteins, what results in the formation of different esters. Kataoka et al. [97] observed that free PhIP under high temperatures reacts with free albumin creating bound HAAs. Thus, it has been hypothesized that HAAs are bound in heat-treated food and thus they can be released under the action of digestive enzymes. This hypothesis was proven by Szterk [10] who treated grilled beef with digestive enzymes (in vitro study). He observed a gradual increase in HAAs content within the passage of a meat sample through each section of the gastrointestinal tract. The increase was observed even though the enzymatic hydrolysis led to partial degradation of proteins and triacylglycerols, what in turn made the biological matrix more complex. Thus, the preparation of samples as well as analysis was way more complicated. Similarly, Chen et al. [98] observed increased concentration of HAAs in smoked sausages after acid hydrolysis. There are only few studies evaluating the impact of selected factors on the accessibility of HAAs from meat subjected to in vitro digestion. Kulp et al. [99] simulated an artificial digestion tract in three locations: mouth (amylase), stomach (pepsin), and small intestine (pancreatin) (as in Figure 2) to determine the percentage release of free HAAs from cooked skinless chicken breast meat and top round “London broil” steaks. They have noted that both amylase and pepsin did not degrade bound HAAs. In turn, pancreatin significantly increased content of free HAAs in meat samples up to 6.6-fold. The bioaccessibility of HAAs was dependent on its polarity. Highly polar compounds were released more efficiently (2.4-fold increase for MeIQx; 3.6—IFP; 6.4—PhIP; and 6.6—DiMeIQx). In the case of low polar carcinogens such as PhIP, major improvement in accessibility (52%) after reduction of particle size of meat made before digestion was observed. Another factor affecting the final content of free HAAs in digested samples was the concentration of pancreatic enzymes. A higher number of enzymes favored HAAs release. Furthermore, increased doneness of meat contributes to lower HAAs bioaccessibility. Shrinkage in well-done meat causes stronger binding of HAAs with meat proteins. Nonetheless, the lower release of carcinogens in that meat did not mean the lower exposure to those compounds because well-done meat is characterized overall by a higher content of HAAs. The meat type also has a great impact on the rate of carcinogens release during digestion. A higher release of MeIQx and DiMeIQx was noted for beef in comparison to chicken meat. However, the cause for these differences is unknown. The authors hypothesized that it is related to the composition differences (fat and protein content ratio) between those types of meat. A modified version of the above-mentioned study was conducted by Kim et al. [100]. In the mentioned study, the researchers built an artificial gastrointestinal digestion tract similar to the previous that simulated the mouth, stomach, small and large intestines but they additionally introduced an agar-based solution of two bacteria *Escherichia coli* and *Lactobacillus sakei* separately or as a mixture of both at the stage of intestinal digestion. The criterium for selecting bacteria for study was their domination in microflora. Both chosen bacteria covered a major share of more than 400 species of microorganisms in the human digestive system. What is interesting, the researchers observed the significant decrease in both heterocyclic aromatic amines’ and nitrosamines’ concentrations already at the stage of stomach digestion. Even higher reduction in HAAs content was observed after digestion in the large intestine with enterobacteria for HAAs and with each bacterium (enterobacteria, *L. casei*, and a mix of both) for nitrosamines. The authors did not propose a clear mechanism behind the decrease in HAAs level after in vitro digestion, but they hypothesized a few possible options instead. The first is based on the rapid change in pH between each section of the digestive tract, especially in the case of the stomach. Salvia in the mouth has a pH of about 6.8, but in the stomach the pH drops drastically to 1.5–3 and then is elevated tremendously by the bile salts to the level of 7–8. Bianchi et al. [101] proved that the degradation of HAAs is triggered by low pH (2.8). The second theory implies that bacteria may decrease the content of carcinogens by binding HAAs (especially lactic acid bacteria) or reducing their content with their own enzymes. For instance, *E. coli* contains two nitrite reductases and siroheme-dependent reductase which degrade nitrates to nitric oxide. The effect of pH on HAAs content was also tested by Xue et al. [102]. Based on the obtained data, the authors stated that pH, electrolytes, and sample volume had weak effect on in vitro digestion of HAAs. However, they observed a significant effect of enzyme concentration on the rate of HAAs release after digestion. A double enzyme dose increased free HAAs content by 1.6 times. Moreover, there was no significant change in HAAs level after stomach digestion, suggesting that pepsin does not break bonds between proteins and HAAs. In turn, similarly to previous studies, they observed a higher release of free HAAs after intestinal digestion. However, the release of carcinogens was not equal for each compound ranging from 0.65% for pyridine-PhIP up to 43.84% for β-carbolines. In general, the rate of α- and β-carbolines release was most significant out of all measured HAAs (12.89–43.84%) indicating that these are more capable of forming bound HAAs and are more prone to hydrolyzation. Even more interesting is the second part of their study where the authors added to a digestive model food ingredients such as pepper powder, onions, and apples. The application of pepper powder at 0.5% and 1% did not affect HAAs release, but 1.5% significantly increased free HAAs content (147.72 ng/100 mg SP in the control group vs. 211.87 ng/100 mg SP in 1.5% of pepper powder). In the case of onions, the addition of 100% and 150% elevated the amount of free HAAs, while 50% had no effect. However, apples increased the unbound carcinogens amount in the case of each percentage addition (50%, 100%, and 150%) from 11.16% for 50% up to 54.45% for 150%. The authors stated that the pivotal effect of those ingredients on HAAs content is connected to the occurrence of polyphenols, fiber, and water in them. Those may change the protein structure and enzyme activity as well as exhibit substrates to reactants. It has been proved that polyphenols can directly interact with proteins through covalent bonds and noncovalent interactions, what expands the protein structure giving enzymes full access to bonds what in turn accelerates release of free HAAs [103]. In turn, active substances such as capsaicin in pepper powder were recognized as those promoting activity of pancreatic digestive enzymes [104]. The activity of enzymes may be triggered by various factors including water and fiber content. Thus, the addition of apples and onions rich in these compounds could be partly responsible for increased rate of free HAAs release in the small intestine [102].

## 5. Conclusions

There are many factors affecting final content of carcinogens in heat-treated meat. However, the impact of those was to date almost exclusively researched based on the measurement of free compounds. Reviewed studies underline that the in vitro digestion has a significant impact on the final content of carcinogens accessible for absorption in the small intestine. The degree of HAAs release during digestion depends on various coexisting factors such as the occurrence of bacteria and selected food ingredients in digestive models as well as the number of enzymes added. Thus, the review underlines the need for further, more in-depth analyses of the impact of previously researched factors on the content of total carcinogens, applying artificial digestion as an integral step of sample preparation introduced before further chromatographic analysis. The obtained knowledge will be essential to estimate which factors affect the content of carcinogens (in total) most and, thus, it will be possible to select the most effective methods that will reduce the carcinogenic potential of thermally processed meat.

## Figures and Tables

**Table 1:** The combined effect of cooking method and temperature on the final content of HAAs in meat samples.

Meat Type	Parameters	Effect	Reference
Leg and breast of goose	Boiling at 100 °C; grilling, pan frying without fat and oil, pan frying with oil, deep fat frying at 180 °C; oven at 200 °C, microwave (automatic).	The highest HAAs content was measured in breast heated with a microwave (2.20 ng/g) and in boiled leg meat (2.42 ng/g).	[38]
Beef chops	Sous-vide cooking at 75, 85, and 95 °C; pan frying at 75, 85, and 95 °C; boiling in pressure cooker.	The highest total content of HAAs was measured in pan-fried beef chops at the temperature of 95 °C.	[39]
Chicken breast and duck breast	Pan frying with no oil at 180 °C; deep fat frying at 180 °C; charcoal grilling at 200 °C and roasting (oven) at 200 °C.	Charcoal-grilled chicken breast had the highest total amount of HAAs followed by pan-fried and charcoal-grilled duck breast.	[40]
Pork patties	Boiling to internal temperature of 71 and 77 °C; oven-broiling at 177 and 225 °C; pan frying at 177 and 225 °C.	Greatest HAAs formation was observed in pan-fried pork patties. HAAs concentration increased in meat samples with the increase in internal temperature.	[41]
Pork loin	Electric oven cooking at 180 °C; hot air frying at 180 °C and deep oil frying at maximum power (household electric oven).	Highest HAAs content was observed in meat samples subjected to cooking in an electric oven.	[42]
Lamb patties	Roasting at 200 °C; frying at 200 °C; pan frying at 200 °C and stewing in seasonings at 100 °C.	Higher content of HAAs was noted for patties stewed in seasonings in comparison to roasted, fried, or pan-fried.	[31]
Chicken, beef, mutton	Charcoal grilling (200 °C on the meat surface); deep frying at 180 °C; pan frying at 180 °C; roasting at 200 °C.	Charcoal grilling and deep frying generated higher HAAs formation in comparison to other methods.	[43]
Duck breast	Boiling at 100 °C; roasting at 160, 180, and 200 °C; electric oven at 200 °C; deep frying at 100, 150, and 200 °C; charcoal grilling and microwave cooking (2450 MHz, 700 W).	Pan-fried samples were characterized by the highest final amount of HAAs followed by charcoal-grilled, deep-fried, roasted, microwave-cooked, and boiled.	[44]
Beef and chicken meatballs	Deep fat frying at 150 °C; pan-cooking at 180 °C; charcoal grilling (temperature of 280 °C on meatball surface); oven roasting at 180 °C.	Charcoal grilling was responsible for the highest HAAs generation in beef meatballs while pan frying for chicken meatballs.	[45]
Beef patties	Steam roasting at 100 °C; infrared grilling at 180, 200, and 220 °C; charcoal grilling and microwave cooking using powers of 1000 and 500 W.	The highest content of HAAs was measured in charcoal-grilled beef samples.	[46]
Lamb patties	Charcoal grilling at 450–500 °C; infrared grilling at 240 °C and superheated steam roasting at 240 °C.	Charcoal grilling generated the highest rate of HAAs formation in lamb patties followed by infrared grilling and superheated steam roasting.	[47]

**Table 2:** Factors affecting heterocyclic aromatic amines content and/or mutagenic activity of meat samples or designed laboratory systems.

Factor	Parameters	Effect	Reference
Temperature	100 °C	HAAs not formed for most compounds.	[27]
Temperature	150 °C	HAAs formation at relatively low level.	[29]
Temperature	200 °C	Rapid increase of HAAs formation.	[29]
Time	Time increase from 1 to 2 h	Threefold increase in 4,8-DiMeIQx content.	[48]
Time	3 min and longer	The concentration of HAAs increased during the time of cooking.	[9]
Cooking method	Deep frying, roasting, pan frying, grilling	Highest content of HAAs for deep frying followed by roasting, pan frying, and grilling.	[32]
Cooking method	Broiling, deep frying, pan frying	PhIP was formed in broiled meat in a quantity of 0.07 ng/g, in pan-fried of 0.04 ng/g, and in deep-fried of 0.02 ng/g.	[35]
Cooking method	Grilling, microwave heating, deep frying	Grilled samples had higher content of HAAs in comparison to microwaved and pan-fried.	[36]
Precursor content	Creatinine	In beef flavors with a low content of creatinine fewer HAAs were observed in contrast to beef flavors with a high content of creatinine characterized by the highest content of HAAs.	[53]
Precursor content	Sugar to amino acids ratio	Sugar content higher than in natural state leads to lower formation of HAAs in meat.	[55]
Precursor content	Glycogen content	Reduced content of HAAs in meat obtained from pigs with the RN allele (higher glycogen content) in comparison to normal pigs.	[56]
Meat type	Beef, pork, chicken	Highest content of PhIP was noted in broiled chicken fillet (480 ng/g), lower content for grilled ground beef patties (50 ng/g), and the lowest for grilled pork steaks (28.26 ng/g).	[57] [58] [59]
Fat content	Fat percentage	Higher content of fat decreased production of mutagenic compounds. In beef with 30% fat content 150,000 revertants/kg of fresh beef were detected while that containing 15% of fat 230,000 revertants/kg.	[61]
Fat content	Oxidized fat	The addition of oxidized soybean oil increased PhIP formation as well as addition of lipid oxidation products such as ω-6- and ω-3-derived lipid hydroperoxides, 4,5-epoxy-2-alkenals, 2,4-alkadienals, 2-alkenals, 4-oxo-2-alkenals, and 4-hydroxy-2-nonenal.	[62]
Vitamin E	Vitamin E addition (1% and 10%)	The addition of vitamin E at two concentrations 1% and 10% significantly decreased the formation of PhIP in cooked ground beef patties (of 69% and 72%, respectively).	[64]
Vitamin E	Animal supplementation with vitamin E	There was a trend observed that with increasing tissue levels of α-tocopherol meat mutagenicity was reduced.	[65]
Carotenoids	Carotenoid extracts	Tomato carotenoid extracts addition of 1000 mg/kg inhibited formation of MeIQx in 13% and 4,8-DiMeIQx in 5% in a meat juice system.	[66]
Pyridoxiamine	Pyridoxamine addition	Pyrydoxiamine (0.2 mmol power) lowered PhIP, 4,8-DiMeIQx, and MeIQx level in fried beef patties by about 40%.	[67]
Vitamin C	Vitamin C addition	Vitamin C (0.2 mmol power) lowered PhIP, 4,8-DiMeIQx, and MeIQx level in fried beef patties by about 20%.	[67]
Niacin	Niacin addition	Niacin (0.2 mmol power) lowered PhIP, 4,8-DiMeIQx, and MeIQx level in fried beef patties by about 20%.	[67]
Polyphenols	Apple peel extract addition	Addition of 0.3% of apple peel extract on the surface of beef patties reduced formation of MeIQx by 68%, 4,8-DiMeIQx by 56%, and PhIP by 83%.	[68]
Polyphenols	Turmeric	Addition of turmeric (5%) significantly inhibited norharman and harman formation (by 49.56% and 94.8%, respectively).	[69]
Polyphenols	Red pepper	Addition of 1% of red pepper reduced HAAs formation from 75% up to 100% depending on the compound.	[70]
Polyphenols	Pure phenolic compounds (apigenin, luteolin, kaempferol, quercetin, genistein, naringenin, phlorizin, EGCG)	Epigallocatechin gallate (EGCG), phlorizin and quercetin are the most effective in both reduction of total HAAs (55–70%) and PhIP (60–80%) content.	[71]
Polyphenols	Wine, beer	The addition of wine to marinades for beef sample marination prior to pan frying decreased HAAs formation of 72.5%. In the case of beer, a 25.9% reduction of HAAs content was observed.	[72]

## Data Availability

The data presented in this study are available on request from the corresponding author.
